# Chicago Veterinary Society

**Published:** 1898-01

**Authors:** James Henderson

**Affiliations:** Secretary


### CHICAGO VETERINARY SOCIETY.

ON November 3, 1897, the Society held a joint meeting with the Chicago State Veterinary Association, under the management of the latter. The attendance was very large, there being a number of gentlemen present who were interested in the milk business. Dr. Henderson read a very interesting paper on "Milk Inspection," and Dr. A. H. Baker also read a very interesting paper on "Tetanus in the Horse."

The meeting on November 4th was also a joint meeting, it taking the place of our regular meeting, conducted by our own officers. The chief feature of the meeting was an exposé by Dr. Joseph Hughes of the humorous methods in vogue at the Chicago horse-auction mart. He related the difficulties encountered by a veterinarian who is commissioned to buy horses in the "bull-ring." The veterinarian, it seems, is forced to buy in this ring, because it is the interest of the commission man that it should be so. Purchase in most cases, by private bargain is impossible, because these men as a rule have in most instances advanced money to the shippers to assist them in getting their stock into market. The former in this manner gain control of the disposition of horses so shipped. He criticised the legend which is hung up in the ring, called "serviceably sound." This is applied to horses which, on account of some blemish or defect, are not sound, but which still are supposed to do a reasonable amount of work without the said defect interfering with their usefulness in this direction. He said the term would be more correct if stated "Unsound, but serviceable." In speaking of the terms applied to these defects by the sellers in the ring he mentioned "speck in the eye," which may mean anything from a speck to a loss of vision; "a full hock' includes abnormally large formation, bog spavin, thoroughpin, and bone spavin." "Rounded hock" is the term applied to a curb. "A pea splint" may mean an exostosis of any size, and a "small sidebone" means a calcified cartilage of any size." "Strong in the pastern" means a ringbone of any size,'' and, finally, "crest fallen," but he did not define that, and the members are still guessing. The tests allowed to the purchasers after buying the horses are lamentably inadequate for the purpose of making a satisfactory examination of them, and all sorts of tricks are resorted to in order to mislead the examiners.

The discussion which followed was largely directed to the relative importance of sidebones, which, some of the members thought, if the sidebones had completed their formation and still left the horse going sound, they were justified in advising their clients to buy if the price was proportionate on that account. Others, including Dr. Hughes, thought that the existence of sidebones, to any extent whatever, constituted an unsoundness which justified a veterinarian in absolutely condemning the horse's purchase. As a result of the discussion the following motion was carried by the meeting:

"That the President appoint a committee of not less than five members to draft a guide which will aid us in distinguishing a sound from an unsound or serviceably sound horse when under professional examination, and present the same at our next regular meeting."

James Henderson, M.R.C.V.S.,
Secretary.

